# A Half-Century Cholera Controversy

**Published:** 1896-05-02

**Authors:** 


### Medical Progress and Hospital Clinics.

[*The Editor will be glad to receive offers of co-operation and contributions from members of the profession. All letters should be addressed to* The Editor, at the Office, 428, Strand, London, W.C.]

### A HALF-CENTURY CHOLERA CONTROVERSY.

The story of a medical controversy, told in bald language, is not necessarily very entertaining, though it can hardly fail to furnish much food for thought to the reflective mind. Medical reviewers and editors of medical newspapers should read a little book like Sir George Johnson's "History of the Cholera Controversy."* It is well calculated to make all such both penitent and humble. In the year 1849, forty-seven years ago, Dr. George Johnson, then a young man, had his earliest experience of cholera and its treatment. His first case was the wife of one of the porters of King's College, who was about forty years of age. The woman, who had up to that time been in perfect health, was suddenly seized with vomiting, purging, and cramps. She was, however, warm, and had a good pulse. She was suffering from a mild attack of cholera. Dr. Johnson prescribed for her the routine treatment of the period, namely, Dover's powder. The medicine was designed to check rapidly the discharges from the bowel. In about three hours the young doctor visited his patient again. She was then in full collapse, and died in a few hours. The doctor was, as he says, "horrified," as well he might be, by such a circumstance.

It was not surprising that the mind of a young physician, trained in the best science of the time, should be profoundly moved by the sudden fatal termination of what appeared to him to be a very mild attack of illness, and the entire failure of treatment to check its fatal progress. With the utmost keenness he watched cases placed under the care of other physicians. He saw the same process repeated time after time; a patient attacked by a disease, but apparently in no immediate danger, was treated by certain remedies; and, as soon as those remedies had had time to produce their known physiological effects, the patient was in a state of collapse, from which, in many cases, he straightway passed to death. Assuming that this is a correct statement of facts, it is obvious that a mind endowed with the smallest possible amount of critical capacity could not fail to make at least these two observations: First, that so far as appearances went, the remedy did not improve the patient's general condition, whatever it might do in the way of checking the diarrhœa; and, secondly, that the less satisfactory condition of the patient, with the ultimate fatal termination, not only followed upon the treatment, but seemed to be due to it. No critical mind, we say, could fail to note at least these two very obvious and very undeniable circumstances.

Much thinking brought the young physician to the conclusion that the checking of the diarrhœa was one of the causes of the fatal collapse. But how? He ultimately arrived at the theory that the checked alvine discharges were to some extent reabsorbed by the blood, that they set up septic physiological changes which produced a spasmodic contraction of the pulmonary arterioles, with a consequent gorging of the right heart, and emptying of the left; and that this pulmonary and cardiac check of activity brought oxidation to an abrupt termination, and killed the patient by a form of suffocation, as surely as if he had been drowned or strangled. The post-mortem appearances entirely confirmed this conclusion. Perhaps it would be more correct to say that the post-mortem appearances furnished the data by means of which Dr. Johnson was able to theoretically construct his physiological *modus operandi* of death. If the reasoning thus summarised was then, and is now, correct, then there is no doubt whatever that the astringent treatment of cholera, of which Sir George Johnson is still the resolute opponent, has killed its thousands, and will kill thousands more if persevered in. Clearly, therefore, what Sir George calls the "Cholera Controversy" is a matter of exceeding importance to any country in the world where cholera rages, or where it may, even by a possibility, gain a deadly hold. No population living mainly in large cities, which has fouled rivers and a congested system of sanitation, can afford to laugh at the "Cholera Controversy" as the history of an old squabble, or to regard it as other than a serious topic of discussion, having consequences fraught with the issues of life and death.

Dr. Johnson, carrying his convictions to their logical issue, instituted a treatment the very opposite of that which was orthodox at the time. The alvine discharges in cases of cholera are deadly poisons, he contended. Therefore, to shut them up in the bowel of a patient is to poison him. Get rid of them by all means. Instead, then, of an "astringent" role, with Dover's powder as its instrument, he boldly practised an "evacuant" treatment, of which castor oil was the active agent. And he became satirically known to the writers and leaders of the profession as "Castor Oil" Johnson. The cholera controversy, as thus sketched out, and the history of which has recently been presented to the profession by Sir George Johnson, deals with the question whether cholera is to be treated by the "astringent" or the "evacuant" method; whether, in short, the foul and poisonous excretions of the bowels are to be retained in the body, or are to be cast forth with all possible haste.

In the olden times—that is, in the times when Sir George Johnson was young—there was only one method of deciding such a question, and that was the clinical method. Now, the clinical is an excellent method when used excellently, that is honestly, and with judgment. Unfortunately it lends itself with all too much readiness both to incompetence and to dishonesty. We find, as a matter of fact, in reading Sir George Johnson's book, that by means of the clinical method alone the "astrigentarians" proved their own treatment to be right and that of the "evacuationists" to be deadly, whilst the "evacuationists" demonstrated, on the other hand, that their principles were both scientific and successful, and that the "astrin-

* "History of the Cholera Controversy." By Sir George Johnson, M.D., F.R.C.P., F.R.S. (London: J. and A. Churchill.)

gentarians" were little better than murderers. There the matter stood for many years, and the medical newspapers and reviewers of the time, never so dogmatic as when their knowledge was small, stood by the old and denounced the new, with all the vituperativeness of which combined ignorance and uncriticality were capable. It is that portion of the book which recounts the parts played by medical editors and reviewers that we commend to our professional critics of the present time as a matter for serious meditation.

How stands the cholera controversy—that is, the controversy between the "evacuant" and the "astringent" treatments—to-day? We are in a very different position for deciding the question now from that occupied by Dr. George Johnson and his early contemporaries. Antisepticism and bacteriology have both sprung into existence since 1849. Antisepticism and bacteriology, in our judgment, absolutely settle the question, and settle it in favour of the "evacuation" treatment of Sir George Johnson. No bacteriologist who understands his business would think of abruptly checking the action of the bowels in the early stages of an attack of cholera. His first care would be to get rid of all the cholera bacilli possible by that most natural of all methods, a suitable purge. In like manner, no antisepticist would think of shutting up in the bowels the poisonous exudations from the abdominal blood vessels and lymphatics, any more than he would shut up in the chest, if he could easily avoid it, the exudation of a pleurisy, or in a wound or an abscess cavity the pus which can never by any chance do good, and is certain so often to do harm. But we have greatly outrun our space. The controversy is extremely interesting from every point of view. Every doctor should read it. Read with a philosophic mind, and deliberately, Sir George Johnson's book is like a peep through a large telescope at the whole medical thought of the past fifty years.

